# Annotations

**Published:** 1899-09-02

**Authors:** 


### ANNOTATIONS.

### The Overcrowding of the Underclad.

To the superficial observer one of the most curious facts connected with the overcrowding of the poor is that, so far from objecting to it, they often seem to revel in a close and fetid atmosphere. Medical men, nurses, and district visitors say with one accord that one of the most difficult tasks they meet with is to persuade the poor inhabitants of overcrowded dwellings to open their windows and let in a breath of the fresh air which so plentifully abounds outside, and this is not in great towns alone, where smuts and dirt can be called up as an excuse, but even in the open country. It is cold which the poor dread above all things, and they so huddle together and keep shut the windows for the sake of warmth during the cold months that they become habituated to stuffiness, and thus will not willingly open their windows even in the warmer weather. Overcrowded the poor are; it is a lamentable fact; but there can be no doubt that much of the ill-health and the high mortality which seems endemic in their overcrowded tenements is the result of their habits and customs rather than the necessary consequence of the want of air space. Two hundred cubic feet with an open window is better, so far as purity of air is concerned, than a thousand with the window closed. But the widely open window is not tolerated by those who have not the clothing by which to keep out the cold. All experience, however, goes to show that, if plenty of clothing be worn and the head be wrapped up in a good nightcap, the health is wonderfully improved by sleeping in the open, and that no harm results from the exposure. There are thousands of people in London who habitually pass their nights in an atmosphere which, after the first ten minutes, is definitely poisonous, and unfortunately securing for them a few more feet of cubic space does but little to improve matters. It is good, no doubt, for our sanitary authorities to fix a minimum of cubic space, and to endeavour to prevent more than the proper number being accommodated in each room. But what are 300 ft., or, for the matter of that, what are 500 ft. of space per head at the end of a couple of hours, when every crevice by which ventilation could take place is carefully stuffed up? No amount of cubic space which it would be possible on economical grounds to enforce in the dwellings of the working classes would materially diminish the most urgent evil and the most disastrous result of the overcrowding, namely, that rebreathing of the same air, over and over again, which is at the root of most of the debility and consumption which prevails. We are not great believers in that free distribution of good things among the poor which some look upon as the most saving form of charity, but we are by no means sure that a free loan of unpawnable blankets and nightcaps granted on the one condition that the upper sash was taken out of every bedroom window, would not do more to lessen the fatal results of overcrowding than any other measure which could be undertaken. It is no use preaching open windows among the poor unless blankets and nightcaps are provided. We doubt, however, whether such an offer would be accepted.

### Death Certificates in Coroners' Cases.

A question is continually cropping up as to the duty of the medical practitioner in regard to the giving of a death certificate when a patient dies unexpectedly or as the more or less remote result of an accident. According to law these are cases which should be investigated by the coroner. But also according to law (Births and Deaths Registration Act, 1874) it is required that whenever a registered medical practitioner has been in attendance during the last illness of a deceased person such practitioner shall sign and give to the qualified informant of the death a certificate of the cause of death. Now it is maintained by many coroners, notably by Mr. Braxton Hicks ("Hints to Medical Practitioners concerning the Granting of Certificates of Death"), that the following cases should be referred to the coroner: (*a*) Deaths resulting directly or indirectly from violence, either immediately or remotely; (*b*) doubtful cases, which may be traumatic or idiopathic; (*c*) all cases occurring under suspicious circumstances; (*d*) deaths which may be due to either natural causes, or to neglect or gross carelessness; (*e*) deaths from unknown causes; and (*f*) stillborn children. We quite agree that it is desirable that in all such cases the coroner should undertake an inquiry as to "how, when, and by what means the cause of death was brought about." But all coroners are not like Mr. Braxton Hicks, and it is idle to shut one's eyes to the fact that to refuse certificates in all the cases mentioned above would often result in the practitioner exposing himself to a serious snubbing. Moreover, even if it be the duty of

ANNOTATIONS—*continued.*

the medical practitioner, or of any other person who suspects foul play, to inform the coroner, that does not alter the fact that the practitioner is bound by law to give a certificate so far as his knowledge goes. There is no doubt that the difficulty is one which is not easy of solution. Only recently a case occurred in which a medical practitioner, who had given a certificate in a case of accident according to law, was told by the coroner that doing so was a dangerous practice, and that if anything had been wrong it would have been a serious matter for him. What appears to us to be the simplest way out of the difficulty is for the medical practitioner to comply literally and exactly with the law, refusing the certificate if he does not know the cause of death, but if he does, then filling up the certificate "to the best of his knowledge and belief"; but in all the cases mentioned above writing across the face of it, "This certificate is to be presented to the coroner before registration." If, then, the "qualified informant of the death" is told to take the certificate to the coroner before delivering it to the registrar the practitioner conforms with the law, the coroner is informed of the case (and also is informed of the medical man's opinion about it so far as that goes), and the delay which might be entailed by the mere refusal of a certificate is avoided. Moreover, the document is rendered useless until it has been properly submitted to the coroner. Whenever the time shall come when the law as to the registration of deaths shall be reformed, it is to be hoped that some definite arrangement will be come to by which it shall be rendered obligatory that every death shall be certified either by medical man or coroner. But that is not yet.

### The "Hooligan" Girl.

The "Hooligan" of the male sex has found his way into the police-court, and thence into the newspapers; but less is known of the girl of the same type. We cling to the notion of the gentler sex, forgetting that the gentleness of Bethnal Green might seem rather rough in Kensington or Mayfair. In Bethnal Green itself they know better. The report of the work done by the residents at St. Margaret's House there, and at the daughter settlement in Stratford, shows that the Hooligan girl is a force to be reckoned with. A girls' club has been formed to which the more docile girls of the neighbourhood come. This meets twice a week, when the time is devoted to games and dancing, musical drill, and the like. Part-singing and brush-drawing are also popular with the members and the preparation of a play for performance at Whitsuntide gave interesting occupation for the winter months. But many of the girls of the neighbourhood were too rough to be amalgamated with the others; they would only have spoiled their happy evenings. So a "rough girls' club" was formed. At first it seemed to be almost a pandemonium. The favourite amusements of the members were swinging from the gas-pipes, turning somersaults, fighting, rolling on the floor, and singing songs of an order not suited for the drawing - room, or any other repectable place. When dancing was indulged in the table was preferred to the floor for the enjoyment of it. The ladies in charge of the club had to exercise all their authority to prevent these goings on; and sometimes all their strength to eject incorrigible offenders. By degrees active games were substituted for the earlier amusements, and finally the girls were induced to sit and do needlework while a story was read aloud to them. What a triumph this was can only be known by those who have done similar work. When at length the rough girls quietly enjoyed the play given by the girls of the other club, and even a lecture on Westminster Abbey illustrated by lantern slides, they were well on the way to civilisation. The work done by such settlements of ladies in the poor districts of our large towns can hardly be overrated, nor can the devotion and patience the work demands from those who undertake it be as easily estimated.

### Mr. Malcolm Morris, Tuberculosis, and the Royal College of Physicians.

All credit is due to Mr. Malcolm Morris for the assistance which he has given, in the columns of the *Practitioner*, to the movement for the suppression of tuberculosis. By the energy of his utterances he has done much to compel attention to the subject. We believe, indeed, that he is the inventor of the phrase, "A crusade against tuberculosis," and that he is by way of regarding himself as somewhat of a Paladin, and the *Practitioner* as his right good sword. Thus only can we explain the opinion which he seems to hold that the Royal College of Physicians is jealous of his interference. "It is," he says, "of course, natural that the priests of the tabernacle of tuberculosis should think it sacrilege that a profane hand has dared to touch the ark of their covenant. But one may, perhaps, venture to remind them that action of some kind was needed on the part of someone, and they would not themselves stir a finger. Why? I suppose they had to consider their dignity. They were like the magnanimous person who sang—

'I sits with my legs in a brook,
And if anyone asks me for why,
I catch him a crack with my crook;
"'Tis sentiment makes me" says I,'

But while the College of Physicians, *salvâ reverentiâ*, dangled its legs in the brook and nursed its lofty sentiments, a thing which in the public interest ought to be done was left undone. If it is now being done by others, that is wholly the fault of the College itself, which, apparently, forgot in this, as in other instances, that its business is not simply to 'exist beautifully,' according to the pseudo-æsthetic ideal, but to organise victory against disease." Really this is rather a fine break on the part of the *Practitioner*; but evidently the College of Physicians has caught someone a pretty hard "crack" with its "crook."

### The Duties of the Vaccination Officer.

The Local Government Board have given an important decision as to the powers of guardians to restrict or limit the duties to be performed by their vaccination officer. As is well known, the Leicester Guardians declined to appoint an officer. But the Lutterworth Guardians took a different line. They appointed a vaccination officer, but attached a condition to his appointment, namely, that he should only prosecute defaulters under the Vaccination Acts when specially authorised by the Guardians under their common seal. The Local Government Board have now informed the Guardians that they are advised by the law officers that the duties of the vaccination officer are determined by the Vaccination Acts and Order, and cannot be curtailed, restricted, or superseded by any resolution of the Guardians, and that the appointment in the present case is void. It has been decided, we believe, to proceed with the appointment of an officer in accordance with the terms of the Order.

